# Development of Evidence-Based Disease Education Literature for Pakistani Rheumatoid Arthritis Patients

**DOI:** 10.3390/diseases5040027

**Published:** 2017-11-20

**Authors:** Atta Abbas Naqvi, Mohamed Azmi Hassali, Muhammad Tariq Aftab, Syed Baqir Shyum Naqvi, Fatima Zehra, Rizwan Ahmad, Niyaz Ahmad

**Affiliations:** 1 Discipline of Social and Administrative Pharmacy, School of Pharmaceutical Sciences, Universiti Sains Malaysia, Penang 11800, Malaysia; azmihassali@usm.my; 2 Department of Pharmacology, College of Medicine, Imam Abdulrahman Bin Faisal University, Dammam 31441, Saudi Arabia; mtaftab@iau.edu.sa; 3 Faculty of Pharmacy, Hamdard University, Karachi 75270, Pakistan; doctor_naqvi@hotmail.com; 4 Department of Social Sciences, Shaheed Zulfiqar Ali Bhutto Institute of Science and Technology (SZABIST), Karachi 75600, Pakistan; zehra.fatima90@gmail.com; 5 Natural Products and Alternative Medicines, College of Clinical Pharmacy, Imam Abdulrahman Bin Faisal University, Dammam 31441, Saudi Arabia; rizvistar_36@yahoo.com; 6 Department of Pharmaceutics, College of Clinical Pharmacy, Imam Abdulrahman Bin Faisal University, Dammam 31441, Saudi Arabia; niyazpharma@gmail.com

**Keywords:** rheumatoid arthritis, rheumatologic diseases, knowledge, patient education, Pakistan

## Abstract

Rheumatoid arthritis affects 0.5% to 1% of the population globally and is one of the most common causes of disability. Patient education plays a key role in improving treatment outcomes. The purpose of this study was to discuss the process involved in designing an evidence-based disease education literature for rheumatoid arthritis patients of Pakistan in Urdu language with culturally relevant illustrations. A study was conducted to develop disease education literature using Delphi consensus, content validity, and patient feedback. A panel of experts comprised of university professors and health care experts, including health practitioners and pharmacists as well as a social scientist, was set up to assess the need. Eight patients were randomly selected and were asked to give their feedback. Their feedback was incorporated in the development process. The entire process was carried out in eight steps. A disease education literature for patients of rheumatoid arthritis was developed and edited in the form of a booklet. The booklet contained evidence-based information that must be provided to patients in both Urdu and English languages with culturally relevant illustrations. The availability of such literature is significant, as it enables the patients to seek knowledge at home at their convenience. This home-based knowledge support is as helpful as any other means of medical care. The developed literature is planned to be used in further studies which will evaluate its impact in improving knowledge of RA patients.

## 1. Introduction

Rheumatoid arthritis (RA) is considered as one of the most common causes of disability after osteoarthritis and gout. It affects around 0.5% to 1% of the population globally [1]. It is a chronic auto-immune disease that affects joints and results in pain, swelling, and inflammation. The swelling of joints causes deformity and limits patient mobility, thereby complicating joint movements. This reduces patients’ productivity as well as quality of life. Evidence from the past indicates a varying prevalence of RA in Pakistan. Figures for the prevalence of RA varied geographically, as literature reported a prevalence of 0.142% to 5.5% in the southern and northern regions of Pakistan, respectively [1]. Recently, a study conducted in a tertiary care unit in the city of Karachi located in the southern region reported a figure of 633 (12.9%) for RA patients out of a total 4900 patients who visited the rheumatology clinic in the hospital. This result revealed that disease burden in this region, which was previously 0.142%, has dramatically increased and was more common in females [2,3].

Studies conducted around the globe report a varying level of disease knowledge among RA patients. In Europe, patients in Estonia had low knowledge about RA; however, the same was significantly higher among Polish patients [4,5]. A study conducted in Finland reported patient knowledge ranging from poor to satisfactory [6]. Patients in the UK exhibited good knowledge about diagnosis and treatment but lacked knowledge related to the adverse effects of drugs [7]. Evidence from the region around Pakistan indicated that the knowledge of Iraqi and Pakistani patients was quite low [8,9].

Keeping in view the nature of the disease and its consequences, it is important to educate patients about the disease and its management at home. Studies have reported that patient-centered education plays a vital role in increasing disease knowledge [7,8,9]. Several studies have been conducted in the UK with the aim of education literature development for RA patients [10]. Some studies incorporated patient perspectives in developing the literature [11]. Studies have highlighted that rheumatoid arthritis requires careful management daily, which patients need to understand to incorporate disease treatment effectively in their lives. This self-management of RA can only be achieved if patients are aware of the disease and its management. Hence, incorporating patient perspectives in literature development was helpful to address patient’s concerns. Such patient-oriented literature demonstrated success in achieving short-term RA treatment goals as well as a long-term increase in patient knowledge [12,13]. Studies conducted in the USA and Switzerland also highlighted that education about the disease can have a significant impact in improving patients’ therapeutic outcomes [14].

A study conducted in Pakistan highlighted the need for customized education and awareness programs for RA patients [9]. Since the progression of the disease is slow, patients usually are unaware and do not visit rheumatology clinics unless the pain becomes unbearable. This low disease awareness has the potential to become a barrier to treatment of RA among Pakistani patients [9]. This knowledge deficit may be reduced by providing patient-centered disease knowledge. A study conducted in the UK evaluated the impact of a rheumatoid arthritis disease education leaflet on patient knowledge. It reported that the customized education material increased knowledge among RA patients in the first follow-up and concluded that a similar study may be conducted to evaluate the effectiveness of this intervention [13,14]. In congruence with this notion, the purpose of our study was to design evidence-based disease education literature in the form of a patient education booklet available to Pakistani RA patients in the local language, Urdu, with culturally relevant illustrations.

## 2. Methodology

A two-month study was conducted to develop an evidence-based rheumatoid arthritis disease education booklet for Pakistani patients using Delphi consensus, content validity, and patient feedback.

### 2.1. Rationale

The need to develop an evidence-based disease education literature for Pakistani rheumatoid arthritis patients is based on the fact that there is an absolute dearth of educational material available to such patients in Urdu language. Although literature exists on the internet as well as in the form of books, it may present language and cultural barriers. Moreover, the literature sometimes may be too detailed, which becomes overwhelming for patients. Studies conducted in Pakistani RA patients have highlighted that disease management may improve if patients are aware and educated about the disease [2,9].

### 2.2. Target Population

The literature was especially designed for Pakistani patients suffering from rheumatoid arthritis.

### 2.3. Expert Panel

The panel of expert included four university professors from different nationalities and work environments, two rheumatologists working in Pakistani health care sector, and a general practitioner who had practiced medicine in local and international settings. A clinical pharmacist and three community pharmacists with experience in counseling Pakistani patients were also included. Finally, a social scientist was also invited to be a part of the panel. The reason for including experts from a wide range of practice areas was to address all related factors that may play a role in several aspects of disease state management. University professors with a research background of the subject were included to develop evidence-based literature. Rheumatologists and a general practitioner were included to provide a reflective analysis of their personal interaction with RA patients. Similarly, clinical and community pharmacist shared their personal experiences as disease educators for this population. The social scientist provided a theoretical account of patients’ psyches and expectations from a cultural perspective.

### 2.4. Patient Participation

The study included patients in literature development process. A total of eight randomly selected patients were invited to evaluate the literature and give their feedback for each theme on a scale of 1–10. A score of 5 or more was considered satisfactory. The patients were also asked to give their feedback. Randomization was done by inviting every odd numbered (male/female) patient to participate in the study. This hour-long process took place during rheumatology out-patient clinic visiting time in the health care setting. After receiving confirmation of participation from four female patients, the process was modified to include every odd numbered male patient only. This was done to include an equal number of male and female participants, as female patients appeared in greater numbers. Selected participants were given RA literature separately and were asked to provide their feedback at the pharmacy counter in their time of convenience, usually in their next visit or via telephone.

### 2.5. Needs Assessment

Learning objectives identified by the panel were incorporated during literature development. A total of four rounds of focus group discussion using Delphi consensus were conducted. The development process identified objectives such as patients’ education level and interests, language, economic and cultural factors, pre-existing knowledge about disease, and venue of literature use. The literature was developed in such a way that patients who had no formal education could understand the text. To enhance patient interest and overcome language and cultural barriers, the text was supplemented with pictures of similar patients in local dress representative of Pakistani culture. Moreover, the literature was also available in Urdu language to enhance patient understanding. It was hypothesized that the intended audience would have no previous knowledge of the disease. Hence, the literature was developed at a beginner’s level and in a booklet format available free of cost, thereby allowing patients to read it at home and at a time of convenience.

### 2.6. Ethics Approval and Patient Consent

The study was approved by the Research Committee of the School of Pharmaceutical Sciences, Universiti Sains Malaysia (USM) located in Penang 11800, Malaysia (Ref#P-FD0014/17-R) as well as the Institutional Review Board of Clifton Central Hospital (Letter#24082017-2). The study was a part of clinical trials PACTRA (NCT#03254745) and ERADEL (NCT#03336684), which are registered on Clinical Trials.gov [15,16]. All patients gave consent before assessment. They were briefed about the study objectives and were handed the booklet after confirming their participation.

## 3. Results

### 3.1. Development Process

The development process took place in eight steps. The first step was to identify the learning objectives from patient’s point of view. The second step was to conduct a round of discussion. A panel of experts comprising university professors, health care experts, and a social scientist was set up to assess the need for developing evidence-based rheumatoid arthritis disease education literature for Pakistani patients. In the first round of focus group discussion, the panel reflected on their experience with RA patients and discussed patients’ perspectives and expectations. They formulated six core areas to address in literature. These included disease information, pathophysiology, symptoms, epidemiology of RA in Pakistan, common lab tests, and treatment objectives. The literature was developed considering these recommendations.

The panel reviewed the contents in second meeting and further identified areas that patients should know about. These included highlighting importance of medication and treatment adherence as well as dietary and lifestyle modification. This topic was added in the second version of the draft, after which it was piloted in eight patients who were randomly enrolled at the venue. The patients were handed the literature and asked to grade each domain in terms of its usefulness on a scale of 1–10. A score of 5 was considered satisfactory. Moreover, the patients were also asked to give their feedback. The results revealed that all domains were essential; however, patients also sought literature regarding self-care of RA at home. Patient feedback is presented in Table 1.

These findings were discussed in the meeting and, subsequently, text related to self-care techniques in RA was added. The domains finalized in the literature are presented in Table 2 in the order of arrangement.

The fourth round of panel discussion reviewed the literature for readability and clarity of text. The panel graded the respective domains in two categories, i.e., important and necessary, and necessary but not important. The grading was used to calculate the content validity ratio (CVR) for each literature domain as well as the content validity index (CVI). The content validity index for the literature was reported at 0.74 (SD 0.2). A consensus was reached at this point.

### 3.2. Urdu Translation Process

After finalizing the literature, it was then translated into Urdu. In the translation process, the linguistic, technical, and conceptual equivalence was checked, and the translated version was deemed equivalent to English version. This was carried out by two experts in pharmacy and medical subjects, respectively, whose native language was Urdu, and second language was English. After the initial translation, it was verified by handing the translation to an Urdu language expert from a non-health background who back-translated it in presence of the previous two experts. Some conflicts in language constructs and terminologies emerged during this process. The issues were resolved by handing both original and back-translated versions to the same pool of rheumatoid arthritis patients. Difficult constructs and terminologies were then revised based on patient understanding and clarity. The literature in Urdu was validated at this step.

### 3.3. External Review Process

The English and Urdu versions of the disease education literature were finally subjected to an external review by language experts. The literature was then sent to two university professors who belonged to pharmacy and medical backgrounds and had experience working in Pakistani and international health care sectors as well as in academia. The reviewer comments were positive and encouraging. No modification was suggested.

**Figure 1:**
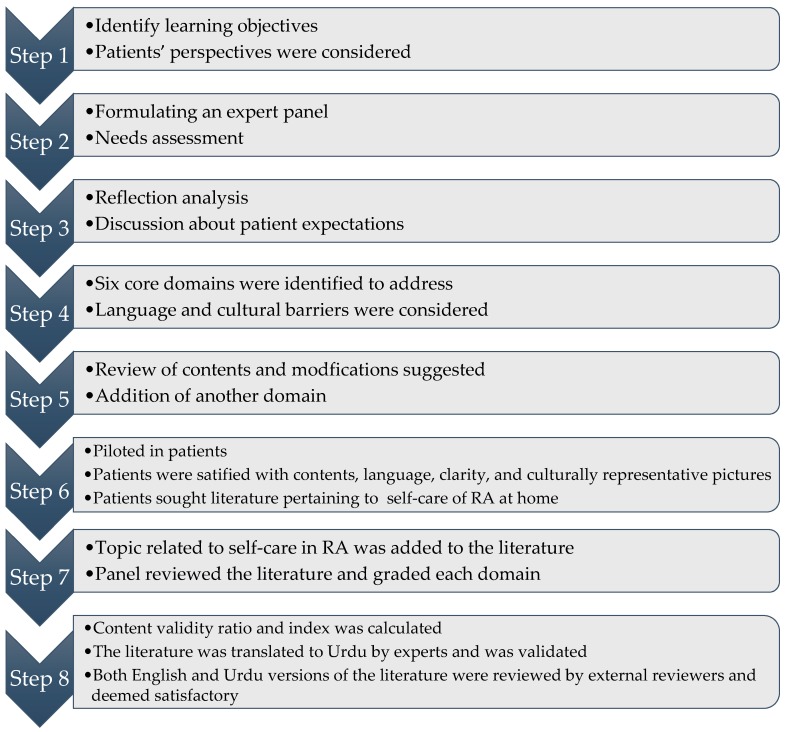
Flowchart of the development process of literature.

The flowchart of development process is presented in Figure 1. The individual responses of panel members toward literature domains is presented in Table 3 and the content validity ratio (CVR) for each literature domain in presented in Table 4.

## 4. Discussion

Rheumatoid arthritis (RA) may slowly progress over time, which sometimes proves difficult for a patient to manage. Many studies conducted in different geographical places of the world have emphasized the need for patient-oriented disease awareness and education [6,8,9]. There is a scarcity of literature concerning RA in Pakistan. Previous studies highlighted a varying prevalence of RA ranging from 0.142% to 5.5% in Pakistan [2]. A recent study established a soaring prevalence of 12.9% in the country. Moreover, a study conducted to document the level of knowledge regarding RA among Pakistani patients reported a low level of disease awareness and education [9].

To address this knowledge deficit among Pakistani patients, an evidence-based patient education literature concerning RA was formulated. It was designed with not only health care providers’ perspectives, but patients’ perception, feedback, and satisfaction were also considered. The process of designing the literature employed Delphi consensus and was completed in eight steps. A panel was set up for this purpose that included professors, rheumatologists, and pharmacists. The results of patient feedback helped the panel to analyze any deficiencies in the literature. Special attention was paid to translate the literature in Urdu language with addition of pictures that are representative of Pakistani audience. The literature was also sent to external reviewers for an unbiased opinion. Further to this, it also underwent content validity.

Unfortunately, Pakistan has a low doctor-to-patient ratio, resulting in a huge patient burden in health care settings. According to the report of the World Health Organization (WHO), the ratio stands at 1:1254 [17]. In this context, physicians are unable to provide detailed information to patients regarding their disease and its management. Therefore, the development of disease education literature becomes vital. Moreover, in many cases patients fail to retain the information provided by doctors over longer durations. This is crucial for chronic illnesses such as RA, since it requires continuous management by patients at home. Hence, the availability of literature is significant, as it enables the patients to seek knowledge in their home and at their convenience. This knowledge support at home is inexpensive and as helpful as any other means of medical care.

Although ample information regarding disease management is available to patients over the internet, many Pakistani patients are unable to utilize these educational resources due to the dearth of literature in the local Urdu language. Hence, there exists a possibility of even educated patients not being able to benefit from the information due to this language barrier. Thus, the provision of linguistically appropriate literature is vital for creating awareness amongst local patients, thereby improving their disease knowledge and treatment outcomes.

Currently, the literature is under trial in Pakistani RA patients and its impact on patient knowledge is being evaluated.

## 5. Conclusions

Evidence-based disease education literature for patients of rheumatoid arthritis was developed. This literature contains updated information that must be provided to patients in a manner that is acceptable to Pakistani patients in both Urdu and English languages, with culturally relevant illustrations. This can serve as an alternative to patient education provided by health care experts and may be helpful for patients in their daily disease management at home. The literature will be available to the public following permission from authors. The process description is in the public domain and may be beneficial to authors conducting similar studies.

## Figures and Tables

**Table 1:** Patient feedback.

S.No	Literature Domains	Mean Score (Minimum/Maximum)	SD
1.	Introduction of disease	8.81 (8/10)	0.923
2.	Pathophysiology	7.63 (5/10)	1.923
3.	Symptoms	8.88 (8/10)	0.641
4.	Epidemiology of the disease in Pakistan	7.63 (6/9)	1.061
5.	Diagnostic tests	9.13 (8/10)	0.835
6.	Treatment and medications	9 (8/10)	0.756
7.	Introduction to pharmacists and their role	6.38 (4/9)	1.685

**Table 2:** Educational domains with the order of arrangement in literature.

S.No	Literature Domains
1.	Introduction of Rheumatoid Arthritis
2.	Pathophysiology
3.	Symptoms
4.	Epidemiology
5.	Diagnostic tests
6.	Treatment and medications
7.	Self-care in Rheumatoid Arthritis
8.	Introduction to pharmacists and their role

**Table 3:** Expert panel response to each domain.

Literature Domains	Expert Panel												
	1	2	3	4	5	6	7	8	9	10	11	12	
	P				R		GP	CP	C			SS	%
1	A	A	A	A	A	A	A	A	A	A	A	A	100
2	A	A	A	A	A	A	A	A	A	B	A	B	83
3	A	A	A	A	A	A	A	A	B	A	A	B	83
4	A	A	A	A	A	A	A	B	A	B	A	B	75
5	A	A	A	A	A	A	A	A	A	A	B	B	83
6	A	A	A	A	A	A	A	A	A	A	A	A	100
7	A	A	A	A	A	A	A	A	A	A	A	A	100
8	A	B	A	A	B	A	B	A	A	A	A	A	75
**Legend**													
A	Important and necessary												
B	Necessary but not important												
P	Professor												
R	Rheumatologist												
GP	General practitioner												
CP	Clinical pharmacist												
C	Community pharmacist												
SS	Social scientist												

**Table 4:** Content validity ratio (CVR).

S.No	Literature Domains	CVR
1.	Introduction of Rheumatoid Arthritis	0.99
2.	Pathophysiology	0.66
3.	Symptoms	0.66
4.	Epidemiology	0.56
5.	Diagnostic tests	0.56
6.	Treatment and medications	0.99
7.	Self care in Rheumatoid Arthritis	0.99
8.	Introduction to pharmacists and their role	0.56
